# Supplementary material for: Design, structure and plasma binding of ancestral β-CoV scaffold antigens
Source: Nat Commun. 2023 Oct 16;14:6527. doi: 10.1038/s41467-023-42200-x (PMC10579346; doi:10.1038/s41467-023-42200-x)
Supplement: Supplementary file 1 — Supplementary Information [file 41467_2023_42200_MOESM1_ESM.pdf]

# Design, structure and plasma binding of ancestral β-CoV scaffold antigens

David Hueting[1,2,#], Karen Schriever[1,2,#], Rui Sun[3], Stelios Vlachiotis[3], Fanglei Zuo[3], Likun Du[3], Helena Persson[2,4], Camilla Hofström[2,4], Mats Ohlin[4,5], Karin Walldén[6], Marcus Buggert[7], Lennart Hammarström[3], Harold Marcotte[3], Qiang Pan-Hammarström[3], Juni Andréll[6,8]*, Per-Olof Syrén[1,2]*

[1]School of Engineering Sciences in Chemistry, Biotechnology and Health, Department of Fibre and Polymer Technology, KTH Royal Institute of Technology, Stockholm, Sweden

[2]School of Engineering Sciences in Chemistry, Biotechnology and Health, Science for Life Laboratory, KTH Royal Institute of Technology, Stockholm, Sweden

[3]Division of Immunology, Department of Medical Biochemistry and Biophysics, Karolinska Institutet, Stockholm, Sweden

[4]Drug Discovery and Development platform, Science for Life Laboratory, Solna, Sweden

[5]Department of Immunotechnology, Lund University, Lund, Sweden

[6]Department of Biochemistry and Biophysics, Science for Life Laboratory, Stockholm University, Stockholm, Sweden

[7]Center for Infectious Disease, Department of Medicine Huddinge, Karolinska Institutet, Stockholm, Sweden

[8]Department of Medical Biochemistry and Biophysics, Karolinska Institutet, Stockholm, Sweden

[#]These authors contributed equally to this work: David Hueting, Karen Schriever

*Corresponding authors e-mail:
juni.andrell@scilifelab.se
per-olof.syren@biotech.kth.se

# Supplementary Information

Table of Contents:


| | | |
|---|---|---|
| **Supplementary Figure 1.** | Full phylogenetic tree used for ancestral sequence reconstruction. | 1 |
| **Supplementary Figure 2.** | Comparison of AnSA sequences to wt SARS-CoV-2 S protein. | 2 |
| **Supplementary Figure 3.** | Expression and solubility of AnSAs compared to HexaPro. | 3 |
| **Supplementary Figure 4.** | Cryo-EM data processing workflow and local resolution estimations. | 4 |
| **Supplementary Figure 5.** | Cryo-EM structure of AnSA-6. | 5 |
| **Supplementary Figure 6.** | Distribution of mutations in AnSA-5 and -6. | 6 |
| **Supplementary Figure 7.** | Thermal denaturation of AnSA-5/6 compared to HexaPro in reference buffer and in presence of chemical denaturants | 8 |
| **Supplementary Figure 8.** | Surrogate virus neutralization assay of AnSAs and HexaPro. | 9 |
| **Supplementary Figure 9.** | Longitudinal tracking of organoid morphology over time. | 10 |
| **Supplementary Figure 10.** | Cell composition of B and T cell types in organoid cultures. | 11 |
| **Supplementary Figure 11.** | Longitudinal tracking of specific antibody production in organoid cultures. | 12 |
| **Supplementary Figure 12.** | Neutralization activity against wt pseudovirus in organoid cultures. | 13 |
| **Supplementary Figure 13.** | Ancestral scaffold antigens harbouring the wt-RBD domain | 14 |
| **Supplementary Figure 14.** | SPR measurements of NTD- and RBD-binding antibody fragments | 15 |
| **Supplementary Figure 15.** | Representative flow cytometry gating strategies. | 16 |
| **Supplementary Table 1.** | Cryo-EM data collection, refinement and validation statistics. | 17 |
| **Supplementary Table 2.** | Demographic data of tonsil donors. | 18 |

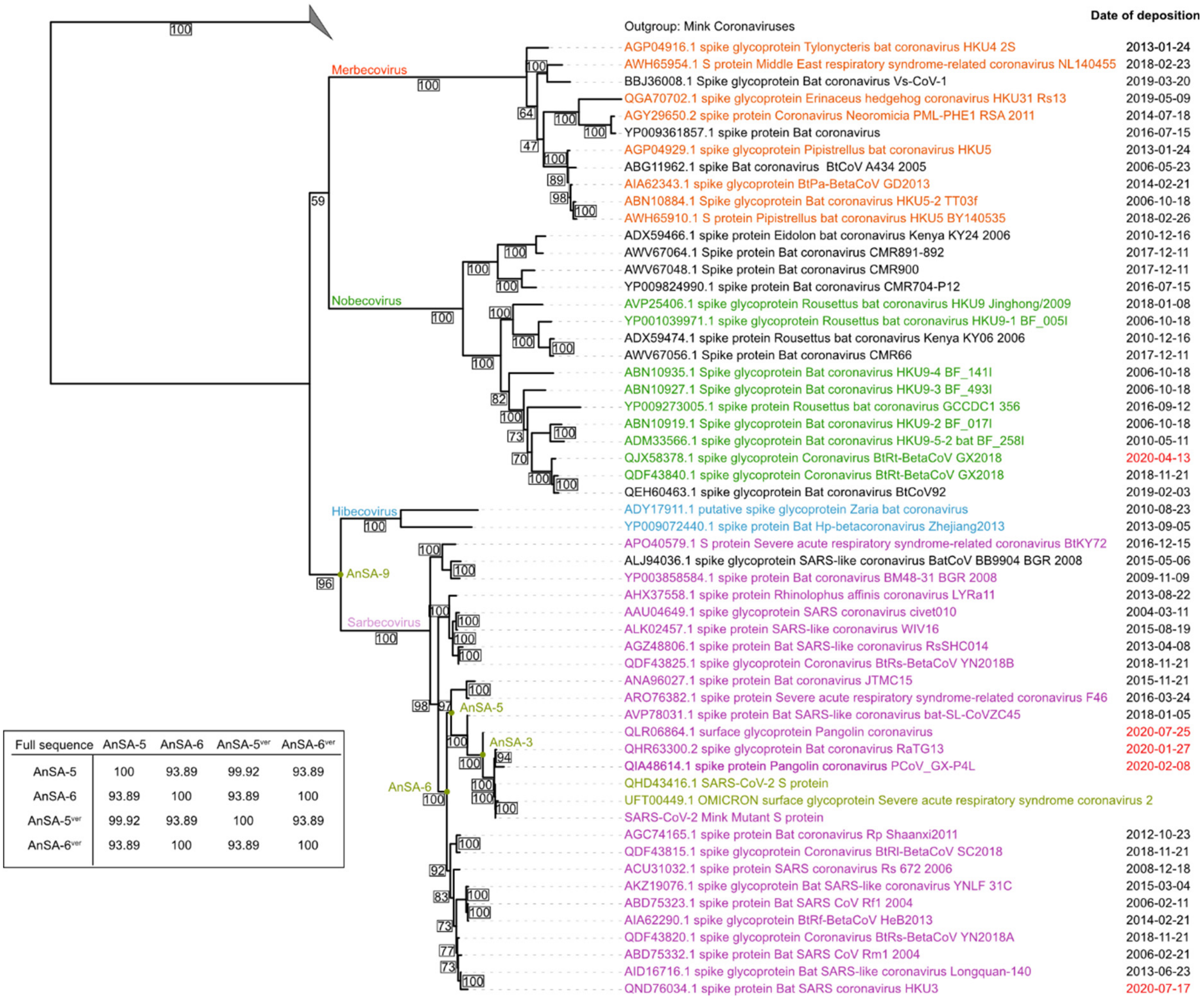


| Full sequence | AnSA-5 | AnSA-6 | AnSA-5^ver | AnSA-6^ver |
|---|---|---|---|---|
| AnSA-5 | 100 | 93.89 | 99.92 | 93.89 |
| AnSA-6 | 93.89 | 100 | 93.89 | 100 |
| AnSA-5^ver | 99.92 | 93.89 | 100 | 93.89 |
| AnSA-6^ver | 93.89 | 100 | 93.89 | 100 |

**Supplementary Figure 1. Full phylogenetic tree used for ancestral sequence reconstruction.** Top, second, third and fourth clade correspond to *Merbecovirus*, *Nobecovirus*, *Hibecovirus* and *Sarbecovirus* sublineages, respectively; sequences that have been assigned to one of these sublineages at deposition are coloured accordingly. Sequences that are not assigned to a sublineage are coloured black. The SARS-CoV-2 S protein sequence is highlighted in light green. The nodes corresponding to AnSA -3, -5, -6, -9 are annotated. The S protein sequence of the omicron variant is shown for reference (light green) and was not used in the reconstruction process. Deposition dates of sequences are indicated on the right. Sequences deposited after 1/1/2020 (red) were considered not to have been available at the onset of the COVID-19 pandemic. AnSA-5^ver and -6^ver were reconstructed using a tree omitting these (red) sequences and their sequence identities to AnSA-5 and -6 (in percent) are indicated in the box.

A

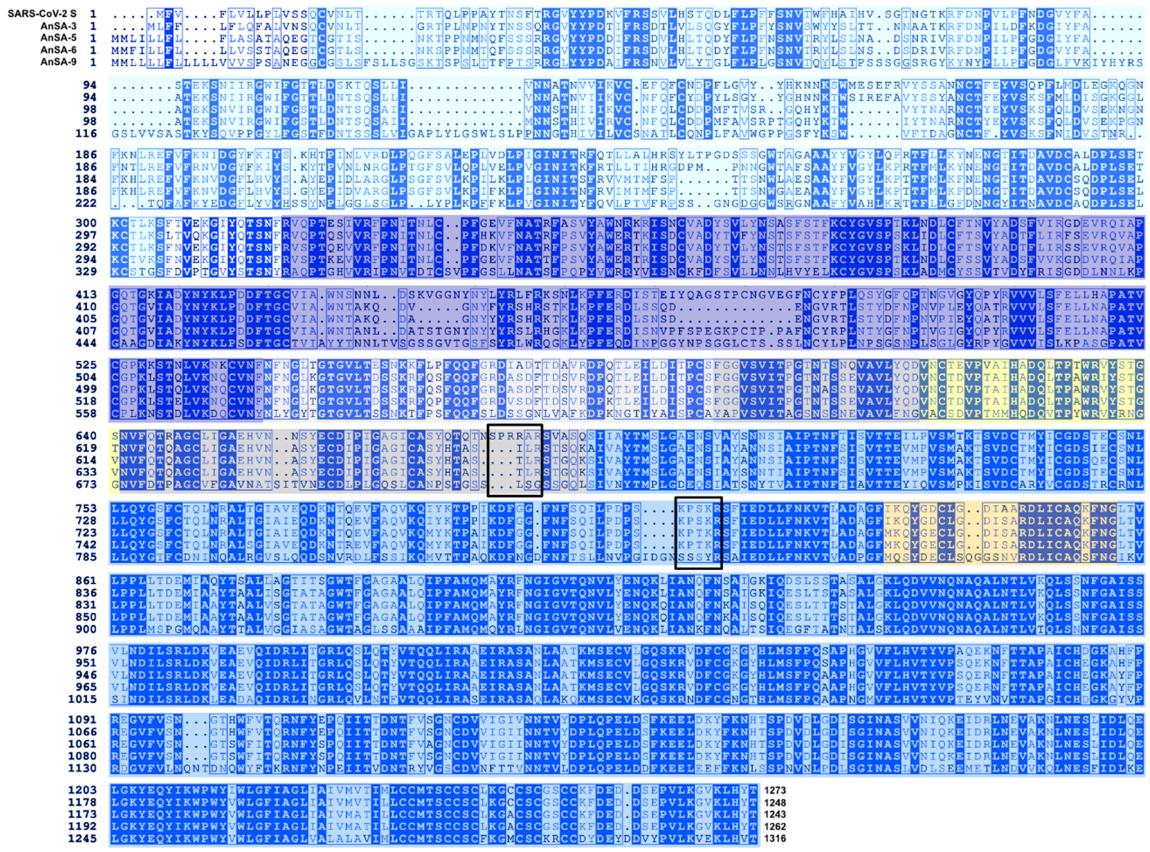

B

| Full sequence | SARS-CoV-2 S | AnSA-3 | AnSA-5 | AnSA-6 | AnSA-9 |
|---|---|---|---|---|---|
| SARS-CoV-2 S | **100.00** | 83.40 | 79.14 | 79.68 | 55.03 |
| AnSA-3 | 83.40 | **100.00** | 87.72 | 83.54 | 55.36 |
| AnSA-5 | 79.14 | 87.72 | **100.00** | 93.89 | 59.00 |
| AnSA-6 | 79.68 | 83.54 | 93.89 | **100.00** | 60.57 |
| AnSA-9 | 55.03 | 55.36 | 59.00 | 60.57 | **100.00** |

| NTD (14-305) | SARS-CoV-2 S | AnSA-3 | AnSA-5 | AnSA-6 | AnSA-9 |
|---|---|---|---|---|---|
| SARS-CoV-2 S | **100.00** | 70.14 | 57.71 | 55.91 | 42.91 |
| AnSA-3 | 70.14 | **100.00** | 65.70 | 61.73 | 44.65 |
| AnSA-5 | 57.71 | 65.70 | **100.00** | 92.25 | 54.18 |
| AnSA-6 | 55.91 | 61.73 | 92.25 | **100.00** | 55.60 |
| AnSA-9 | 42.91 | 44.65 | 54.18 | 55.60 | **100.00** |

| RBD (319-541) | SARS-CoV-2 S | AnSA-3 | AnSA-5 | AnSA-6 | AnSA-9 |
|---|---|---|---|---|---|
| SARS-CoV-2 S | **100.00** | 72.20 | 72.20 | 77.03 | 49.10 |
| AnSA-3 | 72.20 | **100.00** | 94.63 | 78.05 | 47.32 |
| AnSA-5 | 72.20 | 94.63 | **100.00** | 81.46 | 46.83 |
| AnSA-6 | 77.03 | 78.05 | 81.46 | **100.00** | 52.70 |
| AnSA-9 | 49.10 | 47.32 | 46.83 | 52.70 | **100.00** |

| SD1 (542-591) | SARS-CoV-2 S | AnSA-3 | AnSA-5 | AnSA-6 | AnSA-9 |
|---|---|---|---|---|---|
| SARS-CoV-2 S | **100.00** | 80.00 | 78.00 | 80.00 | 50.00 |
| AnSA-3 | 80.00 | **100.00** | 98.00 | 90.00 | 48.00 |
| AnSA-5 | 78.00 | 98.00 | **100.00** | 92.00 | 50.00 |
| AnSA-6 | 80.00 | 90.00 | 92.00 | **100.00** | 52.00 |
| AnSA-9 | 50.00 | 48.00 | 50.00 | 52.00 | **100.00** |

| SD2 (592-690) | SARS-CoV-2 S | AnSA-3 | AnSA-5 | AnSA-6 | AnSA-9 |
|---|---|---|---|---|---|
| SARS-CoV-2 S | **100.00** | 78.95 | 78.95 | 80.00 | 45.83 |
| AnSA-3 | 78.95 | **100.00** | 94.74 | 95.79 | 50.53 |
| AnSA-5 | 78.95 | 94.74 | **100.00** | 98.95 | 50.53 |
| AnSA-6 | 80.00 | 95.79 | 98.95 | **100.00** | 50.53 |
| AnSA-9 | 45.83 | 50.53 | 50.53 | 50.53 | **100.00** |

| S2 (691-1273) | SARS-CoV-2 S | AnSA-3 | AnSA-5 | AnSA-6 | AnSA-9 |
|---|---|---|---|---|---|
| SARS-CoV-2 S | **100.00** | 95.88 | 92.45 | 92.62 | 66.04 |
| AnSA-3 | 95.88 | **100.00** | 95.88 | 95.20 | 66.21 |
| AnSA-5 | 92.45 | 95.88 | **100.00** | 98.80 | 67.58 |
| AnSA-6 | 92.62 | 95.20 | 98.80 | **100.00** | 68.27 |
| AnSA-9 | 66.04 | 66.21 | 67.58 | 68.27 | **100.00** |

**Supplementary Figure 2. Comparison of AnSA sequences to wt SARS-CoV-2 S protein. (A)** Sequence alignment of SARS-CoV-2 S protein and AnSA-3, -5, -6 and -9**.** The NTD (light blue, 14-305), RBD (dark blue, 319-541), SD1 (light grey, 542-591), SD2 (dark grey, 592-690) and S2 (slate blue, 691-1273) domains are highlighted. Two helix/loop motifs in SD2 and S2 domains are highlighted in yellow and orange, respectively. The S1/S2 site and S2' site are highlighted with black boxes. Conserved residues are indicated as bold white letters in dark blue boxes and residues with a similarity score greater than 70% according to ESPript are enclosed by a light blue frame. Figure generated using ESPript v 3.0.[1] **(B)** Full sequence identities and per-domain sequence identities of SARS-CoV-2 S protein and AnSAs (in percent). Domain boundaries are based on wt SARS-CoV-2 S protein.

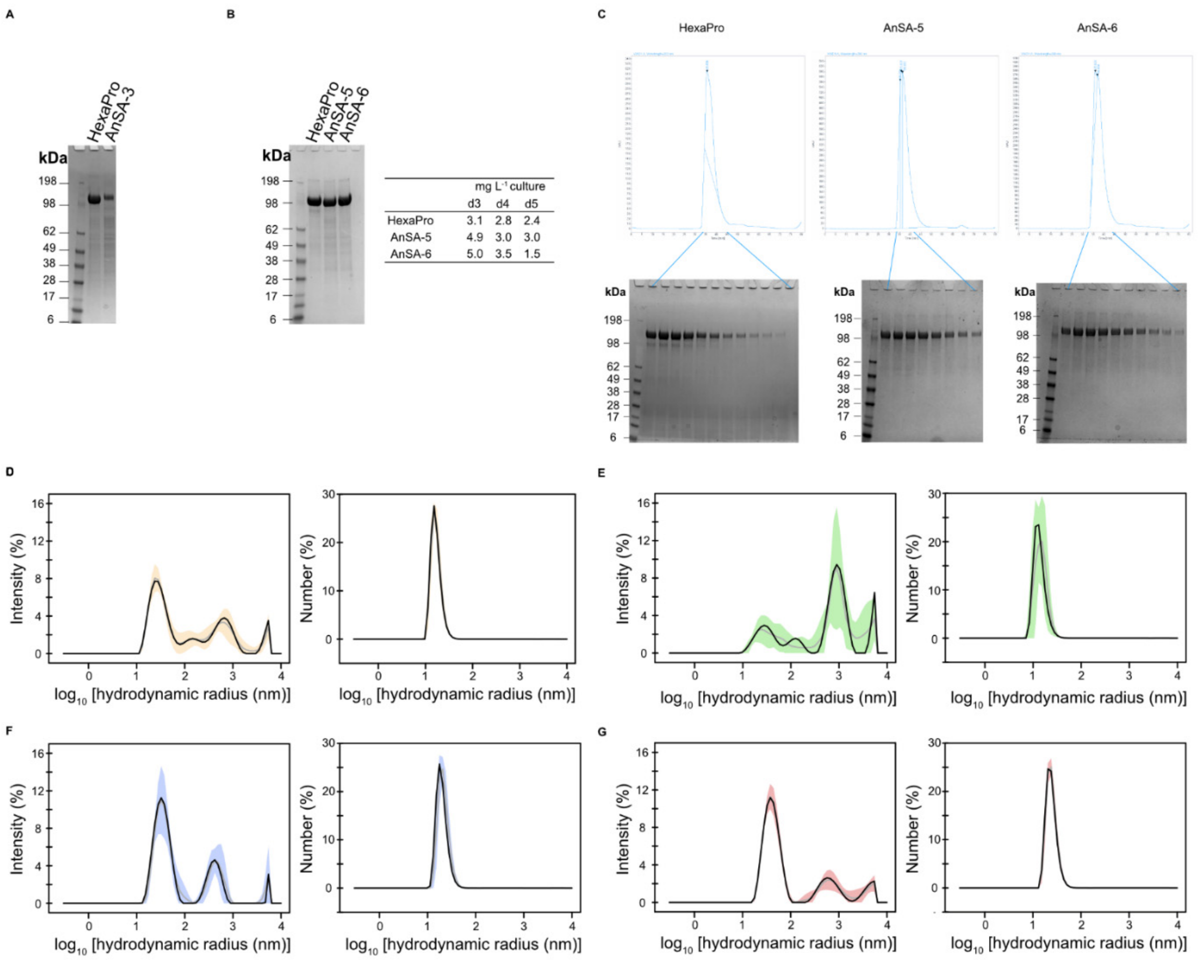


**Supplementary Figure 3. Expression and solubility of AnSAs compared to HexaPro. (A)** SDS-PAGE gel of Ni-NTA purified HexaPro and AnSA-3 obtained from 200 mL of Expi293F culture in 3 days. **(B)** SDS-PAGE gel of purified protein from 300 mL expression culture for HexaPro, AnSA-5 and AnSA-6 after pooling different fractions from size-exclusion chromatography. Expression yields in the table were obtained from 25 mL cell culture transfected for 3, 4 or 5 days, after which the respective culture was harvested, and proteins were purified as described in the Methods section. **(C)** Size exclusion chromatograms (top) and SDS-PAGE gels of individual elution fractions of size exclusion chromatography (bottom) for HexaPro, AnSA-5 and AnSA-6. **(D)-(G)** Left: DLS particle size distributions of **(D)** HexaPro (1.5 mg mL$^{-1}$), **(E)** AnSA-3 (0.4 mg mL$^{-1}$), **(F)** AnSA-5 (1.4 mg mL$^{-1}$) and **(G)** AnSA-6 (1.2 mg mL$^{-1}$) samples plotted by intensity, right: DLS particle size distributions of the same samples plotted by particle number. Average values obtained from technical repeat measurements (n = 5) are plotted as black lines (calculated by ZS XPLORER software) or grey lines (calculated manually from the individual plots), respectively. Standard deviations from technical variation (n=5) are indicated as coloured area plots (yellow – HexaPro, green – AnSA-3, blue – AnSA-5, red – AnSA-6).

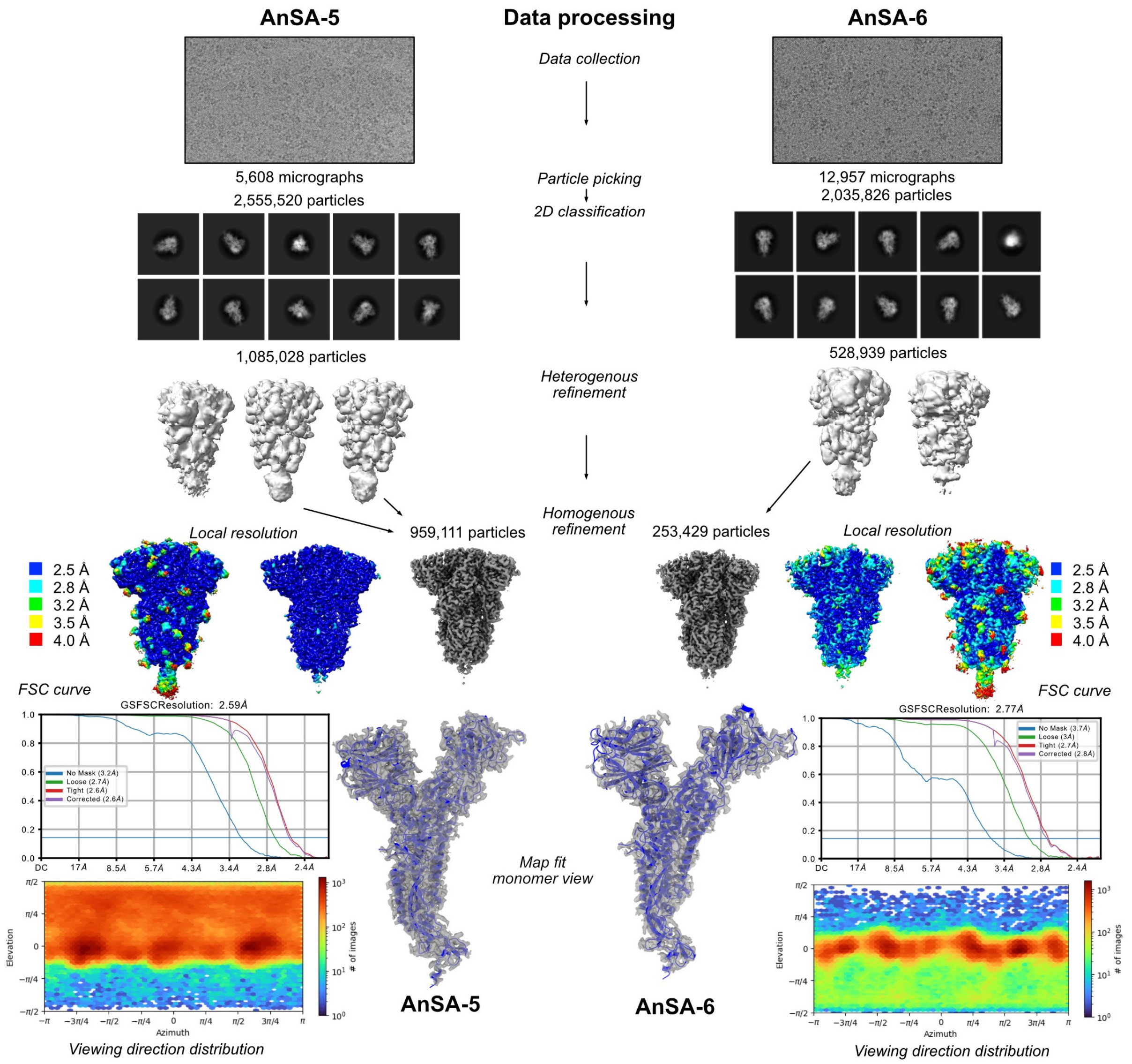


**Supplementary Figure 4. Cryo-EM data processing workflow, local resolution estimations, model map fit, FSC curves and viewing direction distribution Elevation/Azimuth heatmaps.**

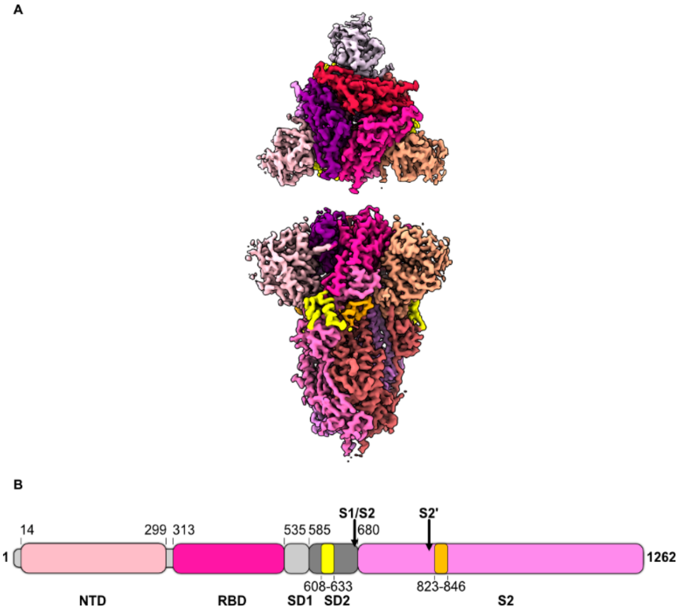

**Supplementary Figure 5. Cryo-EM structure of AnSA-6. (A)** Top view (top) and side view (bottom) of AnSA-6 electron density map generated by cryo-EM. Each one monomer is coloured in pink (monomer A), salmon (monomer B) and magenta (monomer C) with the NTD domains highlighted in lighter shades and the RBD domains highlighted in darker shades. A helix/loop motif in the apex of the SD2 domain is coloured yellow and an adjacent helix/loop motif on the S2 domain is coloured orange. **(B)** Domain overview as bar graph with domain boundaries indicated above the bar. Colours are the same as in (A) and correspond to domain colouring in monomer A. The SD1 and SD2 domains are coloured light and dark grey (grey omitted in (A) for clarity).

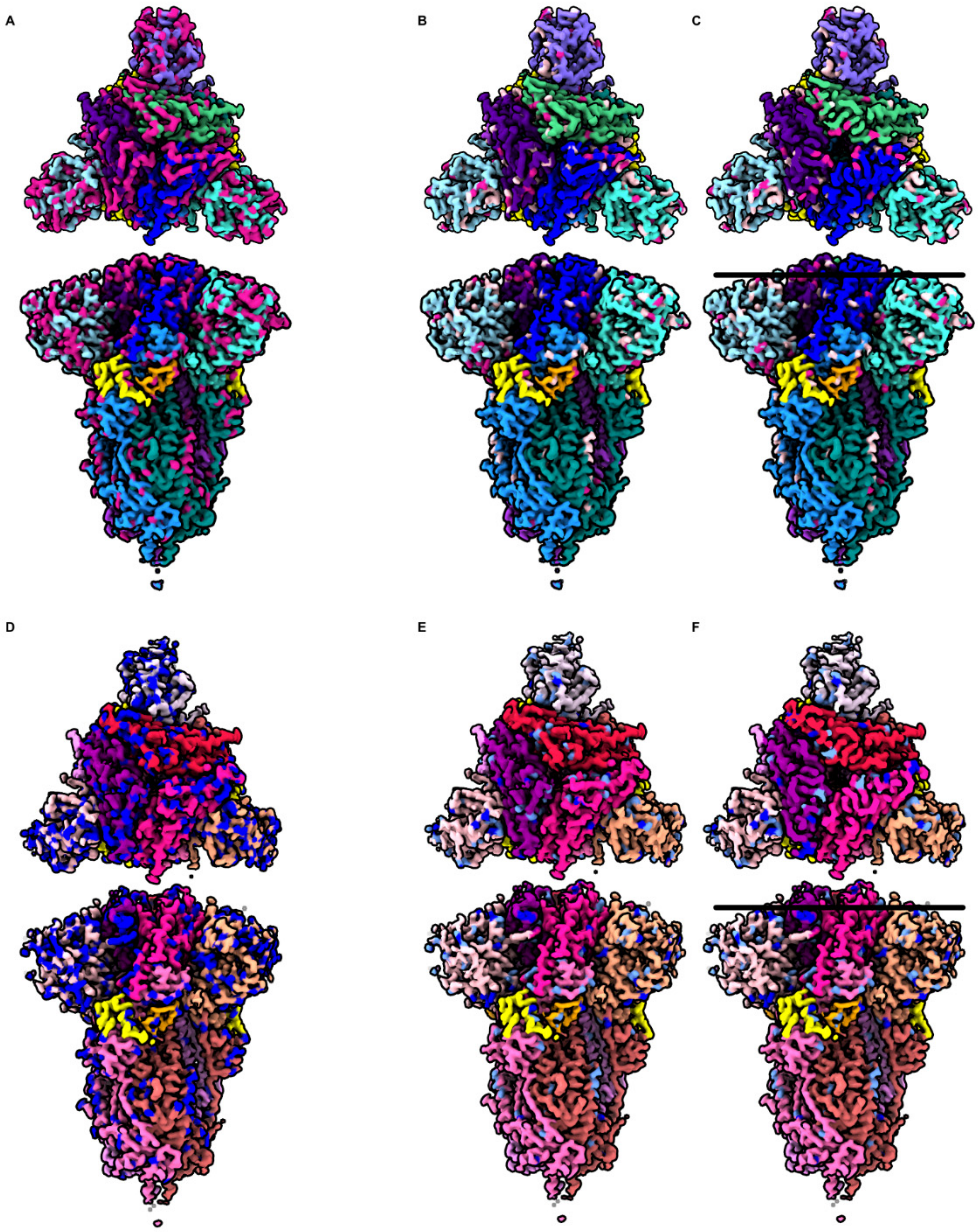


**Supplementary Figure 6. Distribution of mutations. (A)** Top view and side view of AnSA-5 electron density map (colouring as in Fig. 2). All residues that are mutated with respect to the SARS-CoV-2 S protein (Wuhan wt sequence) are highlighted in dark pink. **(B)** Same view as in (A), highlighting mutated residues that are involved in hydrogen bonds. AnSA-5 residues that have non-similar or weakly similar properties to the respective wt residues (according to classification by Clustal Omega) and that form hydrogen bonds via their sidechains are highlighted in dark pink. Residues forming hydrogen bonds via sidechains that have similar properties to respective wt residues or that form hydrogen bonds via their backbone are highlighted in light pink. **(C)** Same view as in (B). The top-view is marginally clipped along the z-axis to show hydrogen bonds at the RBD interface. Approximate clipping height is indicated with a black bar on the side view representation. **(D)** Top view and side view of AnSA-6 electron density map (colouring as in Supplementary Fig. 5). All residues that are mutated with respect to the SARS-CoV-2 S protein (Wuhan wt sequence) are highlighted in dark blue. **(E)** Same view as in (D), highlighting mutated residues that are involved in hydrogen bonds. AnSA-6 residues that have non-similar or weakly similar properties to the respective wt residues and that form hydrogen bonds via their sidechains are

highlighted in dark blue. Residues forming hydrogen bonds via sidechains that have similar properties to respective wt residues or that form hydrogen bonds via their backbone are highlighted in light blue. **(F)** Same view as in (E). The top-view is marginally clipped along the z-axis to show hydrogen bonds at the RBD interface. Approximate clipping height is indicated with a black bar on the side view representation.

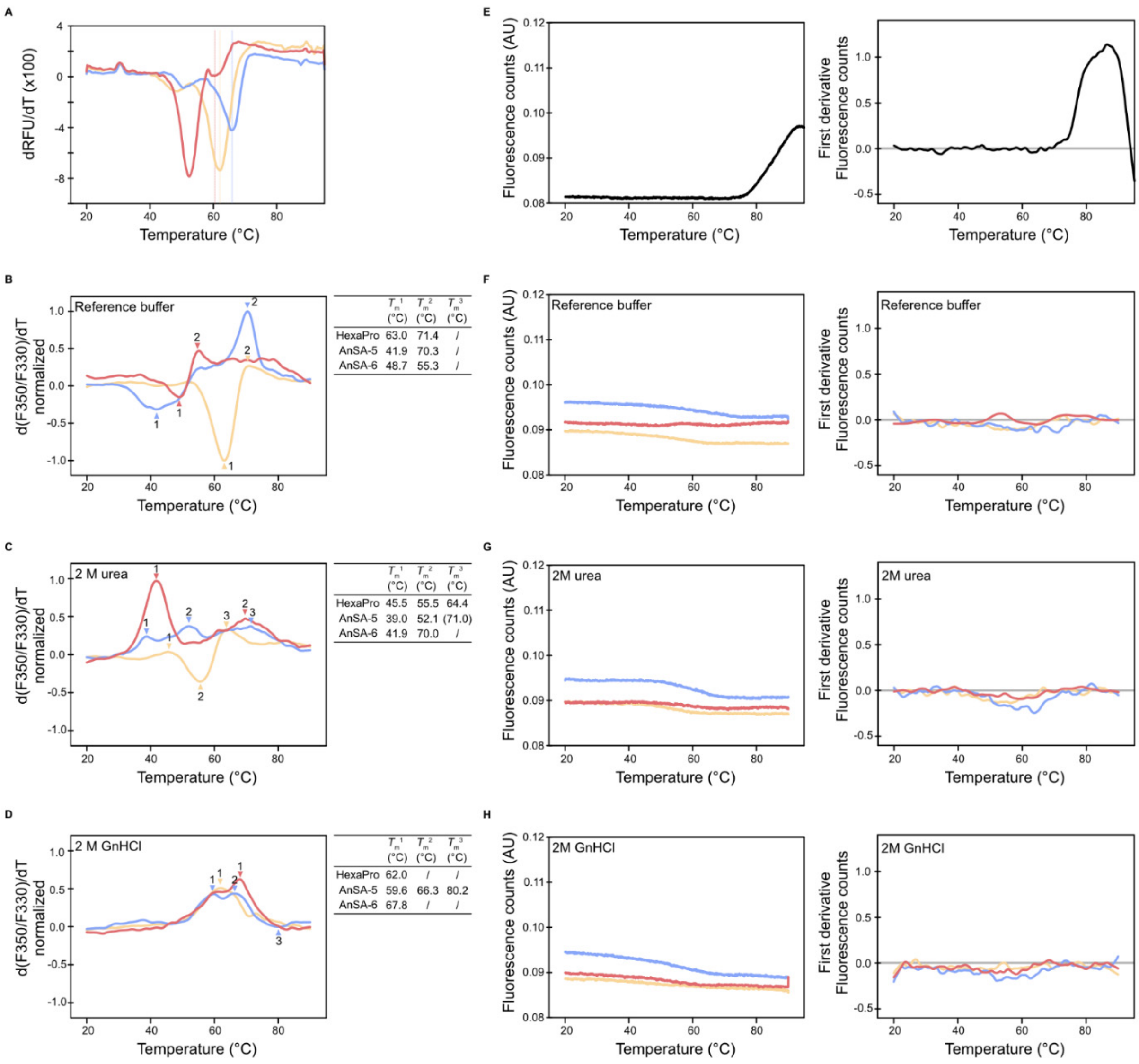


| B | $T_m^1$ (°C) | $T_m^2$ (°C) | $T_m^3$ (°C) |
|---|---|---|---|
| HexaPro | 63.0 | 71.4 | / |
| AnSA-5 | 41.9 | 70.3 | / |
| AnSA-6 | 48.7 | 55.3 | / |

| C | $T_m^1$ (°C) | $T_m^2$ (°C) | $T_m^3$ (°C) |
|---|---|---|---|
| HexaPro | 45.5 | 55.5 | 64.4 |
| AnSA-5 | 39.0 | 52.1 | (71.0) |
| AnSA-6 | 41.9 | 70.0 | / |

| D | $T_m^1$ (°C) | $T_m^2$ (°C) | $T_m^3$ (°C) |
|---|---|---|---|
| HexaPro | 62.0 | / | / |
| AnSA-5 | 59.6 | 66.3 | 80.2 |
| AnSA-6 | 67.8 | / | / |

**Supplementary Figure 7. Thermal denaturation of AnSA-5/6 compared to HexaPro in reference buffer and in presence of chemical denaturants (A)** Thermal unfolding of HexaPro (yellow line), AnSA-5 (blue line) and AnSA-6 (red line) in storage buffer, measured by DSF. Representative results shown (three technical replicates). **(B)-(D)** Thermal unfolding of HexaPro (yellow lines), AnSA-5 (blue lines) and AnSA-6 (red lines) in storage buffer (B) and in storage buffer containing 2 M urea (C) or 2 M GnHCl (D), measured by nanoDSF. Representative results shown (two technical replicates). Different $T_m$ values were determined as local maxima and minima of the first derivative of the 350/330 nm ratios in the nanoTemper software and are indicated in the tables. The values of the first derivative were normalized between -1 and +1 separately for each protein (taking into account all tested buffer conditions). **(E)-(H)** Backscattering traces were recorded to assess aggregation during thermal denaturation for 2 mg mL$^{-1}$ lysozyme in storage buffer (E) and concomitantly to measuring autofluorescence signals for proteins in (B)-(D) in storage buffer (F), 2 M urea (G) and 2 M GnHCl (H). Left: scattering data, right: first derivative of scattering data.

A

| | Severity | Sex | Vaccination | dose | Breakthrough infection (days after the last dose) | sampling date | days after infection |
|---|---|---|---|---|---|---|---|
| Positive plasma no. 1 | severe | M | - | - | - | 2021-03-26 | 24 |
| Positive plasma no. 2 | mild | M | - | - | - | 2021-03-26 | 22 |
| Positive plasma no. 3 | severe | F | - | - | - | 2020-03-13 | 20 |
| Positive plasma no. 4 | mild | M | Pfizer | 2 | 160 | 2022-02-09 | 18 |
| Positive plasma no. 5 | mild | F | Pfizer | 2 | 186 | 2022-02-09 | 38 |
| Negative plasma no. 1 | | F | - | - | - | - | - |
| Negative plasma no. 2 | | M | - | - | - | - | - |

[a] Positive plasma samples 4 and 5 are from individuals vaccinated with two doses of mRNA vaccines followed by breakthrough infection.
[b] Negative plasma no. 1 and 2 are pre-pandemic samples.

B

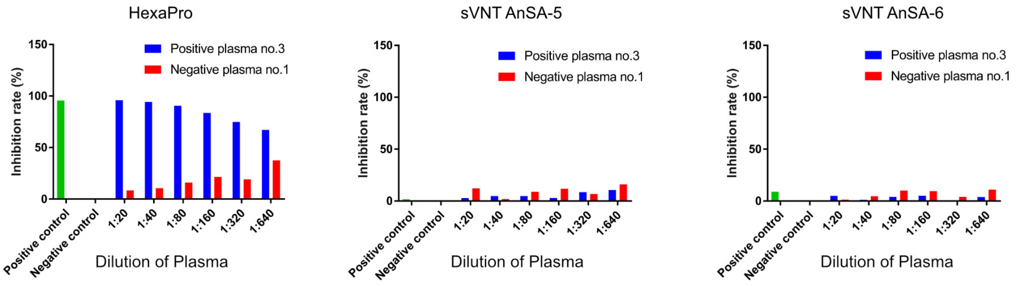


**Supplementary Figure 8. Ancestral scaffold antigens (A)** Demographic and clinical characteristics of the COVID-19 patients (n =5) and negative controls (n =2). The age of donors varies between 37 and 83. **(B)** Surrogate virus neutralization assay of HexaPro, AnSA-5 and AnSA-6. The absence of signal in the positive control for AnSA-5/6 samples indicates that there is no binding of AnSA-5/6 to ACE2. Each column on the graph represents the percentage of inhibition for a single measurement.

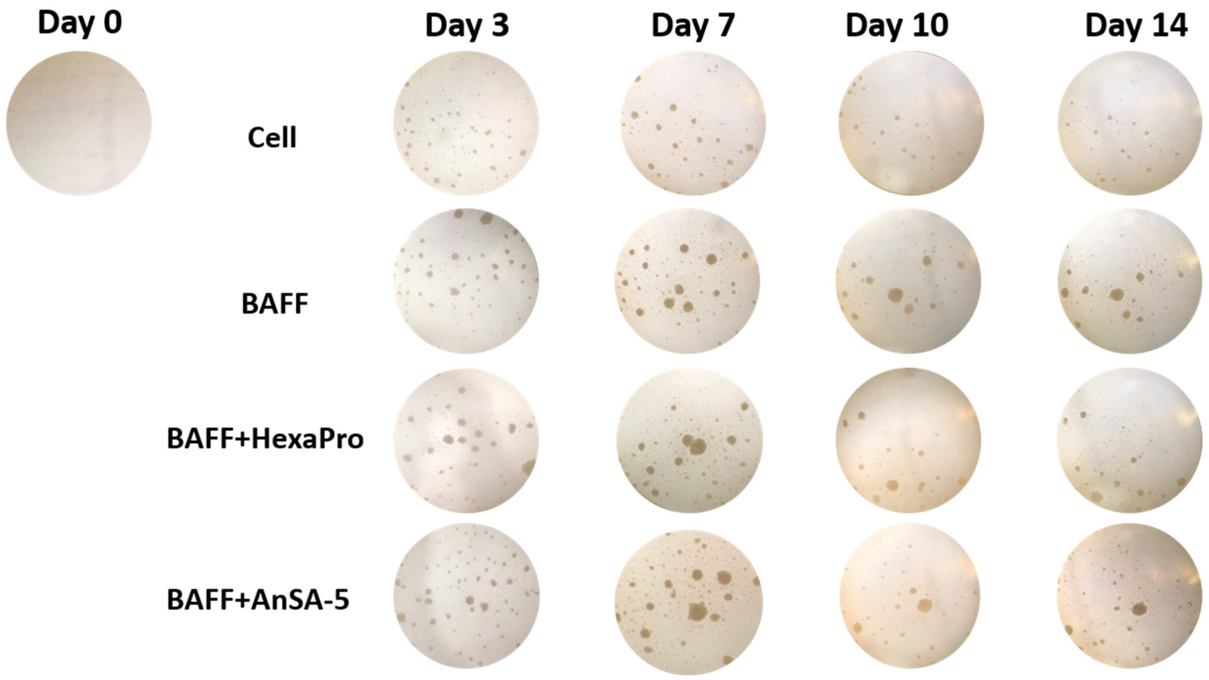


**Supplementary Figure 9. Longitudinal tracking of organoid morphology over time.** Representative stereo-microscopic images of unstimulated and stimulated organoids of donor T147 at days 3, 7, 10, and 14, respectively.

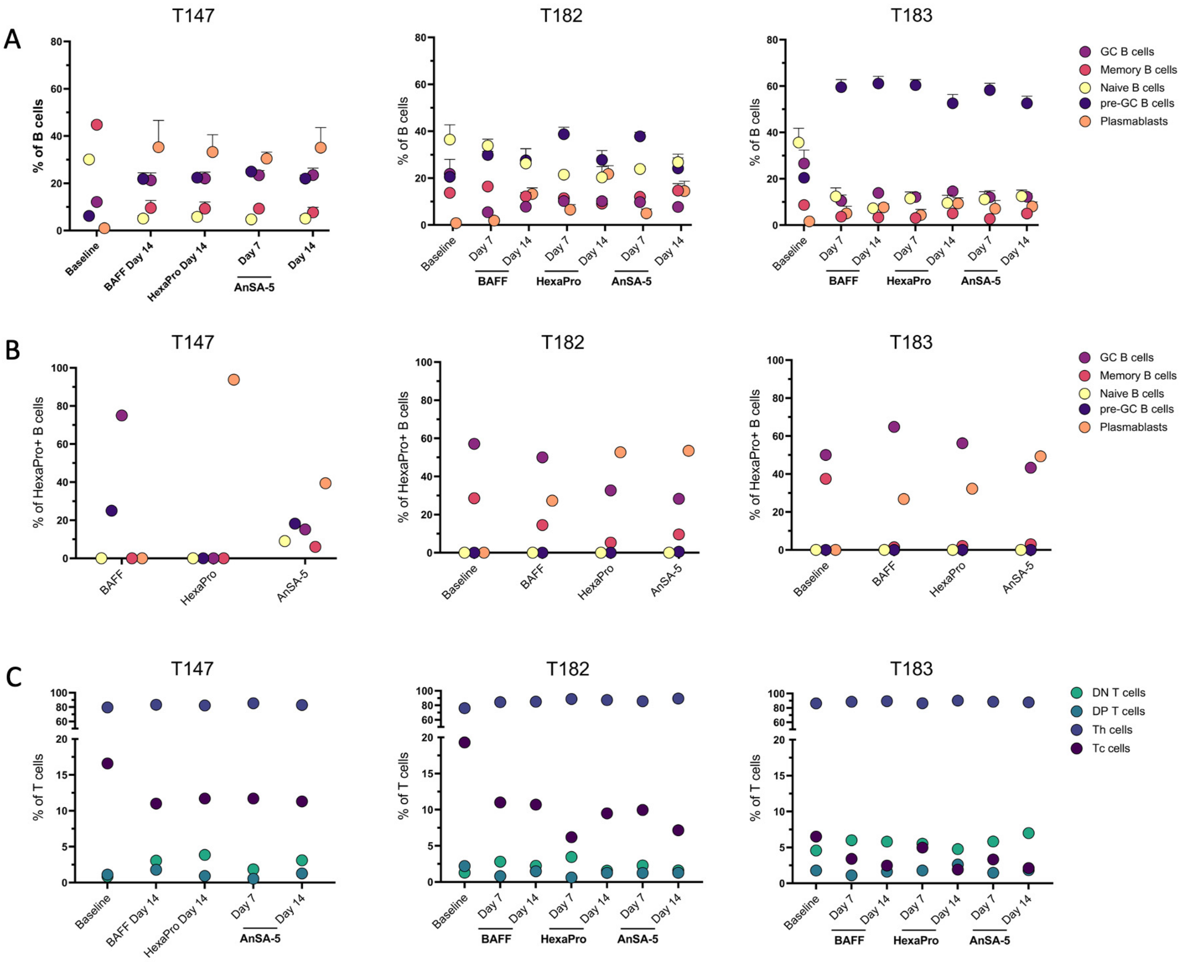


**Supplementary Figure 10. Cell composition of B and T cell types in organoid cultures.** **(A)** Percentages of different B cell populations out of CD19$^{+}$CD20$^{+}$ B cells at days 7 and 14 of the organoid cultures. Error bars indicate mean with the SD of two to three independent measurements of each population with flow cytometry. **(B)** Percentages of different B cell populations out of HexaPro-specific B cells at day 14 of the organoid cultures. (**C**) Percentages of different T cell populations out of CD19$^{-}$ CD3$^{+}$ T cells at days 7 and 14 of the organoid cultures. Baseline values presented in the plots were measured at the beginning of the cultures; HexaPro and AnSA-5 stimulated organoids include BAFF.

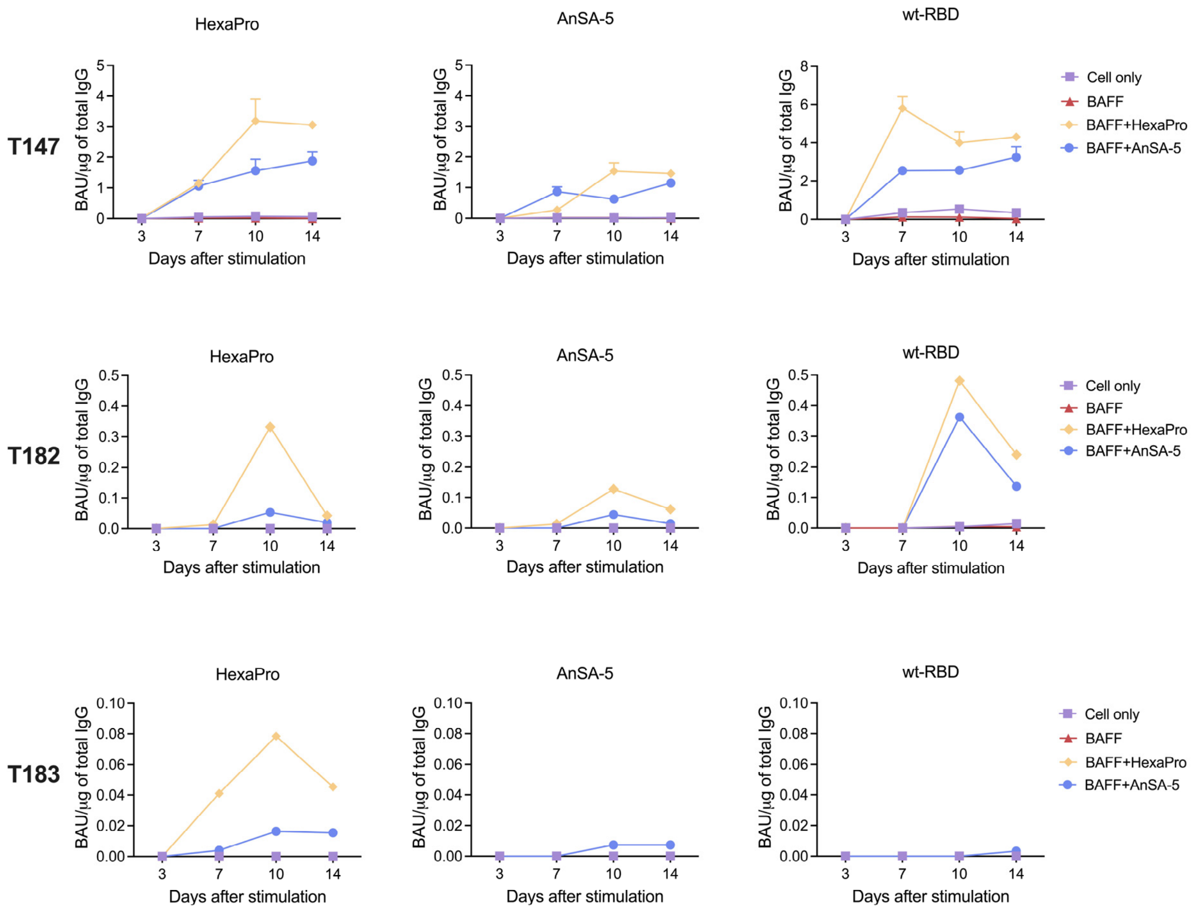


**Supplementary Figure 11. Longitudinal tracking of specific antibody production in organoid cultures.** HexaPro, AnSA-5, and wt-RBD specific IgG levels in unstimulated and stimulated organoid cultures at days 3, 7, 10, and 14, respectively. The results in the graphs are presented as the mean with the standard deviation of duplicate measurements.

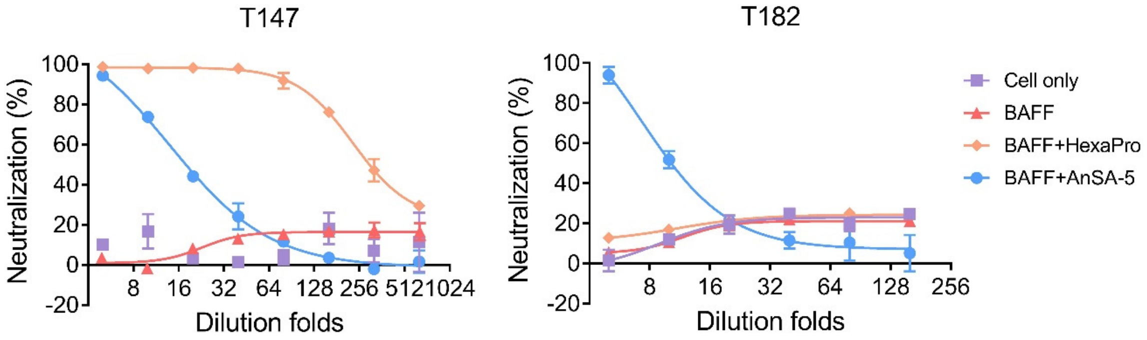


**Supplementary Figure 12. Neutralization activity against wt pseudovirus in organoid cultures.** Two-fold dilutions of day 14 organoid culture supernatants (starting from 1:5 dilution) were tested. Mean ± standard deviation of duplicates for one representative experiment is shown for organoids from donors T147 and T182.

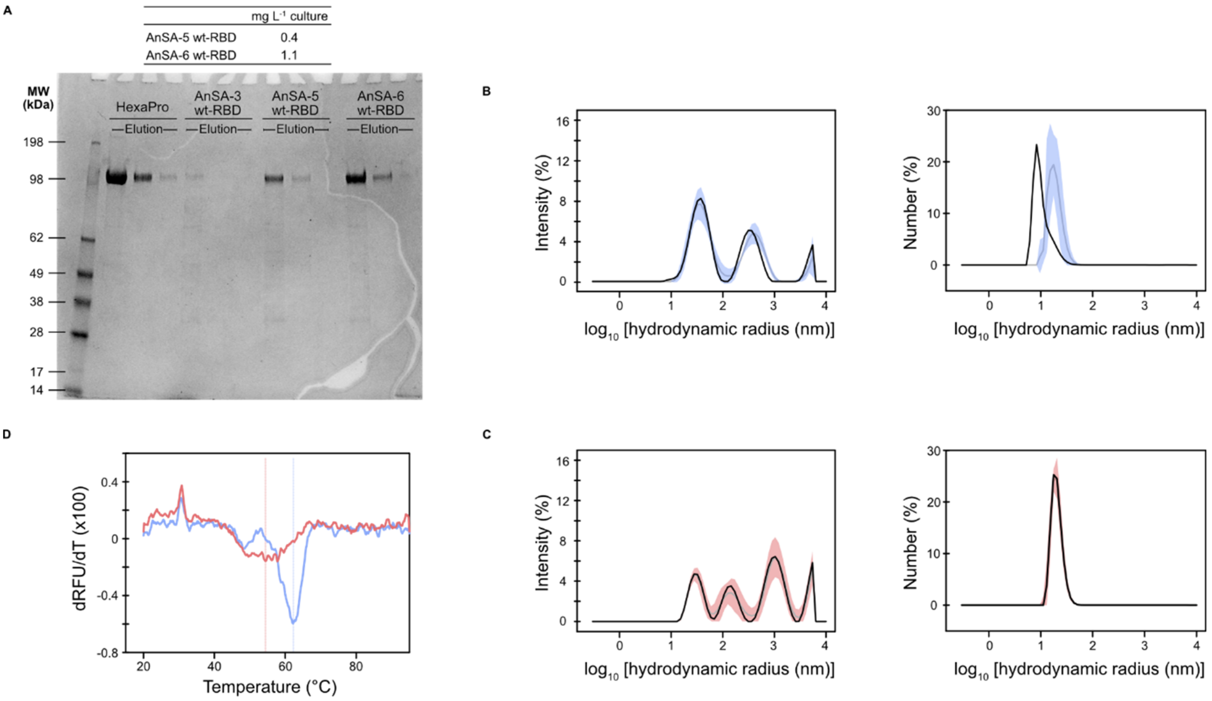


**Supplementary Figure 13. Ancestral scaffold antigens harboring the wt-RBD domain (A)** Test purification of HexaPro, AnSA3 wt-RBD, AnSA-5 wt-RBD and AnSA-6 wt-RBD. Proteins were expressed in 30 mL of Expi293F for 3 days. Elution during Ni-NTA purification was performed in three fractions that were analysed by SDS-PAGE. Yields indicated are from a separate experiment, in which AnSA-5 wt-RBD and AnSA-6 wt-RBD were expressed in 600 mL of Expi293F for 3 days. **(B)-(C)** Left: DLS particle size distributions of (B) AnSA-5 wt-RBD (1.2 mg $mL^{-1}$), (C) AnSA-6 wt-RBD (1 mg $mL^{-1}$) samples plotted by intensity, right: DLS particle size distributions of the same samples plotted by particle number. Average values obtained from technical repeat measurements (n = 4 for AnSA-5 wt-RBD, n = 5 for AnSA-6 wt-RBD) are plotted as black lines (calculated by ZS XPLORER software) or grey lines (calculated manually from the individual plots), respectively. Standard deviations from technical variation (n = 4 for AnSA-5 wt-RBD, n = 5 for AnSA-6 wt-RBD) are indicated as coloured area plots (blue – AnSA-5 wt-RBD, red – AnSA-6 wt-RBD). **(D)** Thermal unfolding of AnSA-5 wt-RBD (blue line) and AnSA-6 wt-RBD (red line) in storage buffer, measured by DSF. Representative results shown (three technical replicates).

| Clone name | Specificity | Hexapro | AnSA-5 | AnSA-6 | AnSA-5 wt-RBD | AnSA-6 wt-RBD |
|---|---|---|---|---|---|---|
| MO176-109 | NTD | 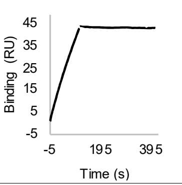 | 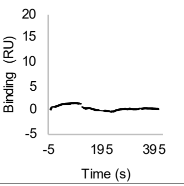 | 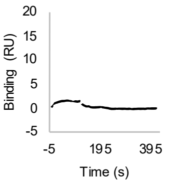 | 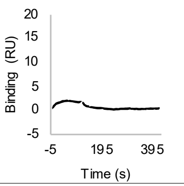 | 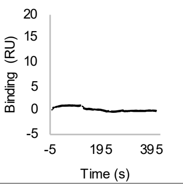 |
| MO176-309 | NTD | 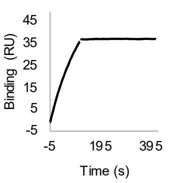 | 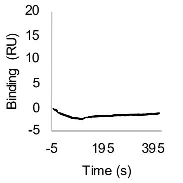 | 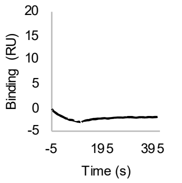 | 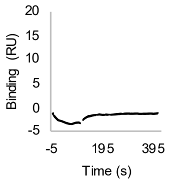 | 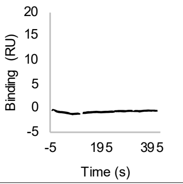 |
| MO176-311 | RBD | 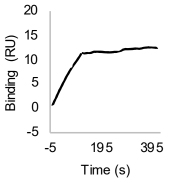 | 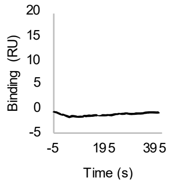 | 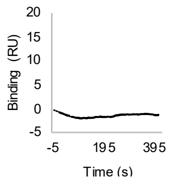 | 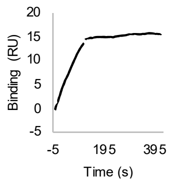 | 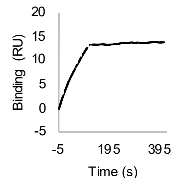 |
| MO176-330 | RBD | 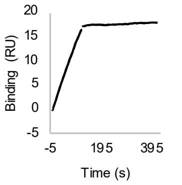 | 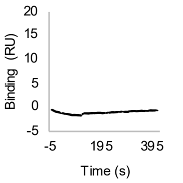 | 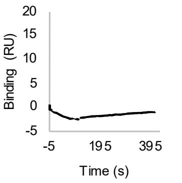 | 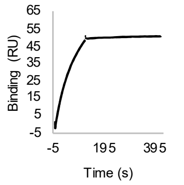 | 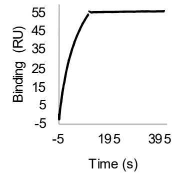 |

**Supplementary Figure 14. SPR measurements of NTD- and RBD-binding antibody fragments.** Binding of antibody fragments MO176-109 (scFv), MO176-309, -311 and -330 (Fab) to antigen constructs HexaPro, AnSA-5, AnSA-6, AnSA-5 wt-RBD and AnSA-6 wt-RBD at 50 nM analyte concentration. Analyte was injected during 120 seconds followed by a dissociation phase.

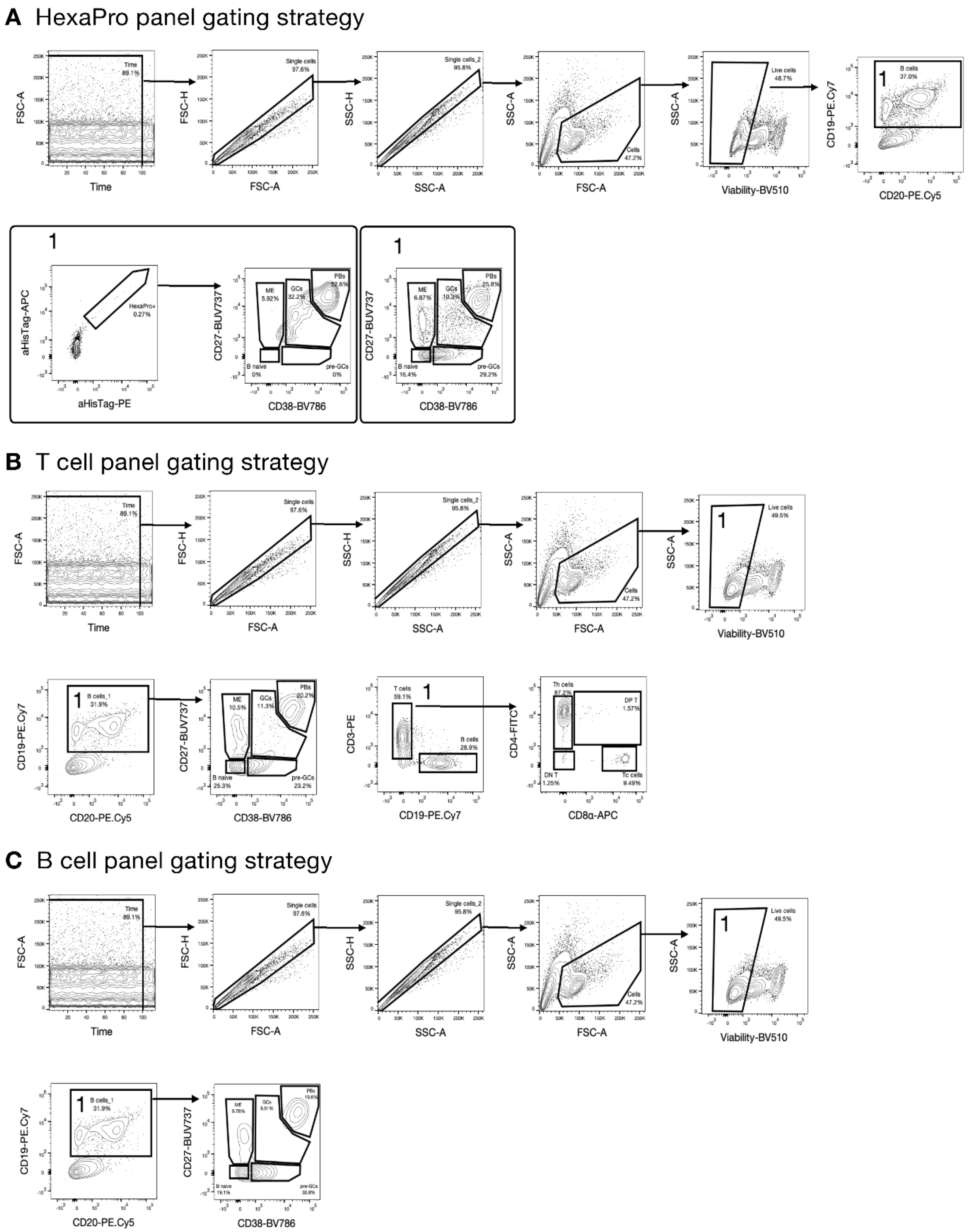


**Supplementary Figure 15. Representative flow cytometry gating strategies. (A)** HexaPro-specific B cell panel gating strategy. **(B)** T cell panel gating strategy. **(C)** B cell panel gating strategy.

**Supplementary Table 1. Cryo-EM data collection, refinement and validation statistics.**

| | #AnSA-5 (EMD-15475) (PDB 8AJA) | #AnSA-6 (EMD-15482) (PDB 8AJL) |
|---|---|---|
| Data collection and processing | | |
| Magnification | 105,000 | 105,000 |
| Voltage (kV) | 300 | 300 |
| Electron exposure (e–/Å2) | 1.11 | 1.11 |
| Defocus range (μm) | -0.6 to -2.0 | -0.6 to -2.0 |
| Pixel size (Å) | 0.833 | 0.833 |
| Symmetry imposed | C1 | C1 |
| Initial particle images (no.) | 2,555,520 | 2,035,826 |
| Final particle images (no.) | 959,111 | 253,429 |
| Map resolution (Å) | 2.6 | 2.8 |
| FSC threshold 0.143 | | |
| Local map resolution range (Å) | 2.5-3.1 | 2.5-3.9 |
| | | |
| Refinement | | |
| Initial model used (PDB code) | PDB 6ZOZ / 7BNN | PDB 6ZOZ / 7BNN |
| Model resolution range (Å) | | |
| FSC threshold 0 (masked) | 2.5 | 2.7 |
| FSC threshold 0.143 (masked) | 2.6 | 2.7 |
| FSC threshold 0.5 (masked) | 2.7 | 3.0 |
| Map sharpening B factor (Å2) | -95.0 | -77.9 |
| Model composition | | |
| Non-hydrogen atoms | 26283 | 26745 |
| Protein residues | 3270 | 3327 |
| Ligands | 57 | 63 |
| B factors (Å2) | | |
| Protein | 16 | 98 |
| Ligand | 24 | 111 |
| R.m.s. deviations | | |
| Bond lengths (Å) | 0.006 | 0.006 |
| Bond angles (°) | 0.764 | 0.693 |
| Validation | | |
| MolProbity score | 1.23 | 1.25 |
| Clashscore | 1.87 | 1.67 |
| Poor rotamers (%) | 0.00 | 0.03 |
| Ramachandran plot | | |
| Favored (%) | 95.95 | 95.26 |
| Allowed (%) | 4.05 | 4.74 |
| Disallowed (%) | 0 | 0 |

**Supplementary Table 2. Demographic data of tonsil donors.**

| Sex | Self-reported COVID-19 infection | Date of infection | Self-reported vaccination | Type of vaccine | Doses | Serum anti-HexaPro IgG (BAU/ml) |
|---|---|---|---|---|---|---|
| M | Yes | 2022 Jul | Yes | BNT162b2 | 3 | 1039.4 |
| M | Yes | 2020 Feb | Yes | NA | 2 | 639.7 |
| M | Yes | 2022 Jan | No | NA | 0 | 714.7 |

NA, not available.

## Supplementary References


1. Robert, X. & Gouet, P. Deciphering key features in protein structures with the new ENDscript server. *Nucleic Acids Res.* **42**, W320-4 (2014).
