# Supplementary material for: Design, structure and plasma binding of ancestral β-CoV scaffold antigens
Source: Nat Commun. 2023 Oct 16;14:6527. doi: 10.1038/s41467-023-42200-x (PMC10579346; doi:10.1038/s41467-023-42200-x)
Supplement: Supplementary file 3 — Description of Additional Supplementary Files [file 41467_2023_42200_MOESM3_ESM.pdf]

Description of Additional Supplementary Files

File Name: Supplementary Data 1
Description: Overview of hydrogen bonds formed by mutated residues in AnSA-5. List of all potential hydrogen bonds in AnSA-5 that involve at least one mutated residue with respect to the SARS-CoV-2 S protein (Wuhan wt), sorted by increasing sequence position of the hydrogen bond donor residue. Hydrogen bonds and saltbridges were identified using the “hbonds”-function in ChimeraX v1.3 with default settings. Secondary structure information for residues (sheet/helix) was obtained from the PDB file of AnSA-5 (chain A) and remaining positions were assigned as loop. The similarity of each residue compared to the SARS-CoV-2 S protein (Wuhan wt) was classified as “none” (red), “low” (yellow), “high” (green) or “conserved” (no colour), according to the classification used by Clustal Omega Multiple Sequence Alignment tool (with the exception that residues A,G,P,V were classified as non-similar to all residues that contain polar side chains). (Sheet1) All identified interactions. (Sheet 2) interactions between sidechain (sc) donor and sc acceptor atoms. (Sheet 3) interactions between sc donor and backbone (bb) acceptor atoms. (Sheet 4) interactions between bb donor and sc acceptor atoms. (Sheet 5) interactions between bb donor and bb acceptor atoms.

File Name: Supplementary Data 2
Description: Overview of hydrogen bonds formed by mutated residues in AnSA-6. List of all potential hydrogen bonds in AnSA-6 that involve at least one mutated residue with respect to the SARS-CoV-2 S protein (Wuhan wt), sorted by increasing sequence position of the hydrogen bond donor residue. Hydrogen bonds and saltbridges were identified using the “hbonds”-function in ChimeraX v1.3 with default settings. Secondary structure information for residues (sheet/helix) was obtained from the PDB file of AnSA-6 (chain A) and remaining positions were assigned as loop. The similarity of each residue compared to the SARS-CoV-2 S protein (Wuhan wt) was classified as “none” (red), “low” (yellow), “high” (green) or “conserved” (no colour), according to the classification used by Clustal Omega Multiple Sequence Alignment tool (with the exception that residues A,G,P,V were classified as non-similar to all residues that contain polar side chains). (Sheet1) All identified interactions. (Sheet 2) interactions between sidechain (sc) donor and sc acceptor atoms. (Sheet 3) interactions between sc donor and backbone (bb) acceptor atoms. (Sheet 4) interactions between bb donor and sc acceptor atoms. (Sheet 5) interactions between bb donor and bb acceptor atoms.
