# Supplementary material for: Design, structure and plasma binding of ancestral β-CoV scaffold antigens
Source: Nat Commun. 2023 Oct 16;14:6527. doi: 10.1038/s41467-023-42200-x (PMC10579346; doi:10.1038/s41467-023-42200-x)
Supplement: Supplementary file 6 — Reporting Summary [file 41467_2023_42200_MOESM6_ESM.pdf]

nature portfolio

Corresponding author(s): Juni Andréll, Per-Olof Syrén

Last updated by author(s): Sep 19, 2023

Reporting Summary

Nature Portfolio wishes to improve the reproducibility of the work that we publish. This form provides structure for consistency and transparency in reporting. For further information on Nature Portfolio policies, see our Editorial Policies and the Editorial Policy Checklist.

Statistics

For all statistical analyses, confirm that the following items are present in the figure legend, table legend, main text, or Methods section.

n/a	Confirmed	
☐	☒	The exact sample size (*n*) for each experimental group/condition, given as a discrete number and unit of measurement
☐	☒	A statement on whether measurements were taken from distinct samples or whether the same sample was measured repeatedly
☒	☐	The statistical test(s) used AND whether they are one- or two-sided<br>*Only common tests should be described solely by name; describe more complex techniques in the Methods section.*
☒	☐	A description of all covariates tested
☒	☐	A description of any assumptions or corrections, such as tests of normality and adjustment for multiple comparisons
☐	☒	A full description of the statistical parameters including central tendency (e.g. means) or other basic estimates (e.g. regression coefficient) AND variation (e.g. standard deviation) or associated estimates of uncertainty (e.g. confidence intervals)
☒	☐	For null hypothesis testing, the test statistic (e.g. *F*, *t*, *r*) with confidence intervals, effect sizes, degrees of freedom and *P* value noted<br>*Give P values as exact values whenever suitable.*
☒	☐	For Bayesian analysis, information on the choice of priors and Markov chain Monte Carlo settings
☒	☐	For hierarchical and complex designs, identification of the appropriate level for tests and full reporting of outcomes
☒	☐	Estimates of effect sizes (e.g. Cohen's *d*, Pearson's *r*), indicating how they were calculated

Our web collection on statistics for biologists contains articles on many of the points above.

Software and code

Policy information about availability of computer code

Data collection	Electron microscopy: EPU (ThermoFisher Scientific)<br>nanoDSF: PR.ThermControl v2.3.1 (NanoTemper Technologies)<br>DLS: ZS Explorer v3.0.0 (Malvern Panalytical)<br>Serum binding and pseudovirus assays: Tecan Infinite 200 PRO microplate reader.
Data analysis	CryoEM Data processing: CryoSPARC v3.3.1<br>Model building: Chimera-X v1.3, ISOLDE v1.1 (Croll et al., 2018), MolProbity (Chen et al., 2010), Coot v0.8.9 (Emsley and Cowtan., 2004), Phenix v1.3 (Liebschner et al., 2019)<br>nanoDSF: PR.ThermControl v2.3.1 (NanoTemper Technologies)<br>Processing of thermal denaturing experiments: Microsoft Excel<br>DSF: BioRad CFX96 manager software (BioRad)<br>Ancestral sequence reconstruction: MEGA-X v10.1.6, IQ-Tree v1.6.12,<br>Cell counter: CELENA® S software 1.1.6<br>Concentration: CFR21 Software (Implen)<br>DLS Data: ZS Explorer v3.0.0 (Malvern Panalytical)<br>Serum binding assay: GraphPad Prism 7.04 software<br>Biacore Insight Evaluation software (Cytiva)<br>Figures: Affinity Designer<br>Image and Illustration: BioRender (BioRender.com)

For manuscripts utilizing custom algorithms or software that are central to the research but not yet described in published literature, software must be made available to editors and reviewers. We strongly encourage code deposition in a community repository (e.g. GitHub). See the Nature Portfolio guidelines for submitting code & software for further information.

Data

Policy information about availability of data

All manuscripts must include a data availability statement. This statement should provide the following information, where applicable:

- Accession codes, unique identifiers, or web links for publicly available datasets
- A description of any restrictions on data availability
- For clinical datasets or third party data, please ensure that the statement adheres to our policy

Additional information or request should be directed and will be fulfilled by Per-Olof Syrén (per-olof.syren@biotech.kth.se) and Juni Andréll (andrell@kth.se)

Materials availability:
Plasmids used in this study are available upon request.

Data availability:
The cryo-EM density maps have been deposited in the Electron Microscopy database (EMBD) under accession code EMD-15475 [https://www.ebi.ac.uk/emdb/EMD-15475] and EMD-15482 [https://www.ebi.ac.uk/emdb/EMD-15482] for AnSA-5 and AnSA-6, respectively.
The atomic coordinates have been deposited in the Protein Data Bank (PDB) under the accession codes 8AJA [https://doi.org/10.2210/pdb8AJA/pdb] and 8AJL [https://doi.org/10.2210/pdb8AJL/pdb] for AnSA-5 and AnSA-6, respectively.

Raw data was uploaded during submission

Human research participants

Policy information about studies involving human research participants and Sex and Gender in Research.

Reporting on sex and gender	The blood and tonsil donors were randomly selected. Information regarding sex and gender was collected from each participant through a questionnaire. Due to the limited number of plasma samples, no gender-based analysis was performed on the binding levels of plasma antibodies to the ancestral scaffold antigens and HexaPro. For the collection of tonsils used to generate the organoid, the three selected donors were male.
Population characteristics	The participants (n=7), aged 37-83, included 4 males and 3 females were randomly selected among cohorts in Sweden and Italy (Sherina et al. 2021, PMID: 33589885; Marcotte et al. PMID: 35018336). The recruitment criteria for collection of blood from convalescent patients included having a documented history of COVID-19 infection (severity and days post-infection) and vaccination (type of vaccine, number of doses, the interval between infection and vaccine doses, days after the latest dose, breakthrough infection), and who were willing and able to provide written informed consent. Five SARS-CoV-2 spike- and RBD-specific antibody positive plasma samples were obtained from convalescent patients including two with breakthrough infection following two doses of the Pfizer vaccine. Negative sera controls were acquired from sera from pre-pandemic two healthy donors<br><br>Whole tonsils and matched blood samples were collected in Sweden from three male individuals (age 4-22) undergoing surgery for obstructive sleep apnea. Tonsil sections from each of the three donor were used for generation of organoids and immunization experiments.
Recruitment	The participants (n=7), aged 37-83, included 4 males and 3 females were randomly selected among cohorts in Sweden and Italy (Sherina et al. 2021, PMID: 33589885; Marcotte et al. PMID: 35018336). The recruitment criteria for collection of blood from convalescent patients included having a documented history of COVID-19 infection (severity and days post-infection) and vaccination (type of vaccine, number of doses, the interval between infection and vaccine doses, days after the latest dose, and breakthrough infection), and who were willing and able to provide written informed consent.<br><br>In addition, whole tonsils were collected from three male individuals (age 4-22) undergoing surgery for obstructive sleep apnea. Matched blood samples were collected to confirm that the donors have been infected or vaccinated by measuring the level of anti-HexaPro antibodies. A written informed consent and self-reported information on COVID-19 infection and vaccination history were obtained from the donors.
Ethics oversight	The sampling of blood was performed under the approval of the Institutional Review Board of Policlinico San Matteo (protocol number P_20200029440) and the ethics committee in Institutional review board in Stockholm (Dnr 2022-00676-01). The collection of tonsils was performed under the approval of Institutional review board in Stockholm (Dnr 2023-02803-01). The participants provided written informed consent before participation in the study. Information regarding age, sex, and history of COVID-19 vaccination and infections was collected from each participant through a questionnaire. The parent or legally authorized representative provided informed consent on behalf of participants under the age of 18 and the requested information for the study. No compensation was provided to participants.

Note that full information on the approval of the study protocol must also be provided in the manuscript.

Field-specific reporting

Please select the one below that is the best fit for your research. If you are not sure, read the appropriate sections before making your selection.

☒ Life sciences ☐ Behavioural & social sciences ☐ Ecological, evolutionary & environmental sciences

For a reference copy of the document with all sections, see nature.com/documents/nr-reporting-summary-flat.pdf

Life sciences study design

All studies must disclose on these points even when the disclosure is negative.

Sample size

The objective was to evaluate whether antibodies elicited against the Spike (S) protein, either by natural infection with SARS-CoV 2 or by combination of vaccination and natural infection, are able to recognize ancestral scaffold antigens (AnSAs). For measuring the level of AnSAs in the serum of convalescent patients, 5 positive plasma samples and 2 controls were used. This was considered sufficient to estimate whether antibodies in blood of convalescent patients can bind to the AnSAs. The AnSAs interacted with plasma of all 5 patients recovered from COVID-19 suggesting that the AnSAs are recognized by plasma antibodies generated by wild-type S protein.

The objective was to evaluate the immunogenicity of AnSA-5 using an established in vitro tonsillar organoid culture model (Wagar et al. PMID: 33432170). Organoids were generated from tonsils of three donors to assess whether AnSA-5 could stimulate an immune response against the S protein of SARS-CoV-2. We demonstrated that AnSA-5 is capable of inducing or boosting a broad-spectrum immune response against the RBD of both wild-type and circulating variants of concern in organoids derived from the tonsils of these three donors, illustrating the potential of AnSAs as antigens for vaccine development.

Data exclusions

No data were excluded.

Replication

All experiments were replicated at least 2 times. If the number of replicates varies from two it can be found in the main text, figure legends and methods.

Randomization

We did not use or report on any randomized data. This was not relevant for the objective of our study which was to show the function of the ancestral scaffold antigens.

Blinding

No data presented was subjected to blinding. We considered that blinding was not relevant at this stage, as the primary objective was to evaluate the function of the ancestral scaffold antigens in in vitro and ex vivo models.

Reporting for specific materials, systems and methods

We require information from authors about some types of materials, experimental systems and methods used in many studies. Here, indicate whether each material, system or method listed is relevant to your study. If you are not sure if a list item applies to your research, read the appropriate section before selecting a response.

Materials & experimental systems

n/a	Involved in the study
☐	☒ Antibodies
☐	☒ Eukaryotic cell lines
☒	☐ Palaeontology and archaeology
☒	☐ Animals and other organisms
☒	☐ Clinical data
☒	☐ Dual use research of concern

Methods

n/a	Involved in the study
☒	☐ ChIP-seq
☐	☒ Flow cytometry
☒	☐ MRI-based neuroimaging

Antibodies

Antibodies used

CR3022 IgG1 (InvivoGen, srbd-mab1)
Flow cytometry staining:
Fixable Aqua Viability dye Thermo Fisher Cat#L34957 Dilution 1:1000
FITC CD3 BD Biosciences Clone HIT3a Cat#561802 Dilution 2:100
FITC CD4 BD Biosciences Clone OKT4 Cat#566802 Dilution 1:100
PE anti-His Tag Biolegend Clone J095G46 Cat#362603 Dilution 5:100
PE CD3 BD Biosciences Clone UCHT1 Cat#555333 Dilution 5:200
PE-Cy7 CD19 BD Biosciences Clone SJ25C1 Cat#341113 Dilution 2:100
PE-Cy5 CD20 BD Biosciences Clone 2H7 Cat#555624 Dilution 2:100
BUV737 CD27 BD Biosciences Clone L128 Cat#564301 Dilution 1:100
BV786 CD38 BD Biosciences Clone HIT2 Cat#563964 Dilution 2:100
APC anti-His Tag Biolegend Clone J095G46 Cat#362605 Dilution 5:100

APC CD8α BD Biosciences Clone SK1 Cat#345775 Dilution 1:100
Human BD Fc block BD Biosciences Clone Fc1 Cat.# 564219 Dilution 1:100
ELISA:
Horseradish peroxidase (HRP)-conjugated goat anti-human IgG (Invitrogen #A18805) Dilution1:15000
Goat-anti-human-igg-unlb (SouthernBiotech #2040-01) Dilution 1:500
Pseudovirus neutralization assay:
IgG anti-RBD antibody SA55 (PMID: 36493787), produced by Genescript, Concentration from 1 μg/ml to 0.01 ng/ml for the standard curve.

SPR:
anti-FLAG M2 antibody (Merck F1804). Dilution to 20 μg/ml and then immobilised onto all 4 surfaces of a CM5 series S chip through NHS/EDC chemistry according to manufacturer's recommendations.
KAPPA antibody (Cytiva 28958325). Diluted to 20 μg/ml and then immobilised onto all 4 surfaces of a CM5 series S chip through NHS/EDC chemistry according to manufacturer's recommendations.

Validation

CR3022 is a recombinant monoclonal antibody (mAb) that was raised specifically against the SARS-CoV and SARS-CoV-2 Spike receptor binding domain and a human IgG1 constant region. The antibody binding has been validated by ELISA using coated spike RBD. https://www.invivogen.com/sars2-spike-cr3022-mab-isotypes

Listed details above is based on the information given by the manufacturer.

Flow cytometry staining:
All antibodies are validated by their respective manufacturers and are quality control tested by surface immunofluorescent staining with flow cytometric analysis. For more information about the antibodies, visit bdbiosciences.com and biolegend.com.

-FITC CD3 BD Biosciences Clone HIT3a Cat#561802:
https://www.bdbiosciences.com/content/dam/bdb/products/global/reagents/flow-cytometry-reagents/research-reagents/single-color-antibodies-ruo/555339_base/pdf/561802.pdf
-FITC CD4 BD Biosciences Clone OKT4 Cat#566802:
https://www.bdbiosciences.com/content/dam/bdb/products/global/reagents/flow-cytometry-reagents/research-reagents/single-color-antibodies-ruo/566802_base/pdf/566802.pdf
-PE anti-His Tag Biolegend Clone J095G46 Cat#362603
https://www.biolegend.com/en-us/products/pe-anti-his-tag-antibody-9861
-PE CD3 BD Biosciences Clone UCHT1 Cat#555333
https://www.bdbiosciences.com/content/dam/bdb/products/global/reagents/flow-cytometry-reagents/research-reagents/single-color-antibodies-ruo/555333_base/pdf/555333.pdf
-PE-Cy7 CD19 BD Biosciences Clone SJ25C1 Cat#341113
https://www.bdbiosciences.com/content/dam/bdb/products/global/reagents/flow-cytometry-reagents/clinical-diagnostics/single-color-antibodies-asr-ivd-ce-ivd/341113_base/pdf/23-5061.pdf
-PE-Cy5 CD20 BD Biosciences Clone 2H7 Cat#555624
https://www.bdbiosciences.com/content/dam/bdb/products/global/reagents/flow-cytometry-reagents/research-reagents/single-color-antibodies-ruo/555624_base/pdf/555624.pdf
-BUV737 CD27 BD Biosciences Clone L128 Cat#564301
https://www.bdbiosciences.com/content/dam/bdb/products/global/reagents/flow-cytometry-reagents/research-reagents/single-color-antibodies-ruo/612829_base/pdf/612829.pdf (This product is the replacement for [564301])
-BV786 CD38 BD Biosciences Clone HIT2 Cat#563964
https://www.bdbiosciences.com/content/dam/bdb/products/global/reagents/flow-cytometry-reagents/research-reagents/single-color-antibodies-ruo/563964_base/pdf/563964.pdf
-APC anti-His Tag Biolegend Clone J095G46 Cat#362605
https://www.biolegend.com/en-us/products/apc-anti-his-tag-antibody-14783
-APC CD8α BD Biosciences Clone SK1 Cat#345775
https://www.bdbiosciences.com/content/dam/bdb/products/global/reagents/flow-cytometry-reagents/clinical-diagnostics/single-color-antibodies-asr-ivd-ce-ivd/345775_base/pdf/23-5031.pdf
-Human BD Fc block BD Biosciences Clone Fc1 Cat.# 564219
https://www.bdbiosciences.com/content/dam/bdb/products/global/reagents/flow-cytometry-reagents/research-reagents/single-color-antibodies-ruo/564219_base/pdf/564219.pdf

ELISA:
https://www.thermofisher.com/antibody/product/Goat-anti-Human-IgG-H-L-Secondary-Antibody-Polyclonal/A18805
https://www.southernbiotech.com/goat-anti-human-igg-unlb-2040-01

Eukaryotic cell lines

Policy information about cell lines and Sex and Gender in Research

Cell line source(s)

Expi293F, ThermoFisher #A14527
293FT, Invitrogen #R70007
293T-hACE2 (a gift from Paul D. Bieniasz, The Rockefeller University)

Authentication

The commercial cell lines were authenticated by the company. hACE2 expression in 293T-hACE2 was shown.

Mycoplasma contamination

The commercial cell lines were tested negative for mycoplasma by the companies. 293T-hACE2 was tested negative upon arrival in our laboratory.

Commonly misidentified lines (See ICLAC register)	No commonly misidentified lines were used.

Flow Cytometry

Plots

Confirm that:

☒ The axis labels state the marker and fluorochrome used (e.g. CD4-FITC).

☒ The axis scales are clearly visible. Include numbers along axes only for bottom left plot of group (a 'group' is an analysis of identical markers).

☒ All plots are contour plots with outliers or pseudocolor plots.

☒ A numerical value for number of cells or percentage (with statistics) is provided.

Methodology

Sample preparation	Tonsil cells were processed fresh on the day of the collection using mechanical dissociation with sterile scalpels and forceps, strained though a 70-µm cell strainer and washed with PBS. Tonsil cells were collected from the organoid cultures on days 7 and 14 from the upper part of the permeable membrane, washed with PBS and filtered through a 70-µm cell strainer before proceeding with the staining protocol.
Instrument	BD FACSymphony A5
Software	FlowJo version v10.8.2
Cell population abundance	No sorting was performed.
Gating strategy	Gating strategies are shown in Extended data Figure 15. Briefly, lymphocytes were gated by standard FSC-A/SSC-A followed by a two-step singlet discrimination (FSC-A vs FSC-H and SSC-A vs SSC-H), and dead cells were excluded using a live/dead dye. B cells were gated by CD19 and CD20 expression while T cells by CD3 expression and no expression of CD19. CD4 and CD8 T cells were identified by CD4 and CD8α staining. HexaPro-specific B cells were defined as the anti-HisTag-PE, anti-HisTag-APC doubled positive population. B cell subpopulations, i.e. Memory (ME; CD27+ CD38-), Naive (CD27-CD38-), Plasmablasts (Plasmablast; CD27+ CD38high), pre-germinal center (pre-GC; CD27-CD38dim/+), and germinal center (GC; CD27+CD38+) B cells were defined based on the expression levels of CD27 and CD38 markers.

☒ Tick this box to confirm that a figure exemplifying the gating strategy is provided in the Supplementary Information.
